# Patient-reported outcomes following treatment of tibial non-union with circular frames

**DOI:** 10.1007/s11751-014-0187-x

**Published:** 2014-02-12

**Authors:** P. Fenton, D. Bose

**Affiliations:** Queen Elizabeth Hospital, Mindelsohn Way, Birmingham, B15 2WB UK

**Keywords:** Tibial non-union, External fixation, Ilizarov, Patient-reported outcome

## Abstract

The management of tibial non-union is challenging with protracted, often arduous, treatments. The purpose of this study was to assess patient-reported outcomes following treatment of tibial non-union in circular external fixators. Twenty-one patients with tibial non-unions who successfully completed treatment at a mean of 10.1 months (range 6–20) in a circular external fixator were sent questionnaires utilising the Enneking scoring system and Euroqol EQ-5D. There were 14 responses. The mean Enneking score was 58.0 % (34.3–77.1). Two patients were enthusiastic about their treatment, while three accepted but would not repeat the treatment. The Euroqol questionnaire found that 8 patients had difficulty with mobility, 10 had difficulty with usual activities and 12 had moderate pain. There was no statistically significant difference in the EQ VAS score of overall health state for treated patients compared with predicted scores for an age- and sex-matched UK population (77.7 vs 83.1, *p* = 0.07).

**Electronic supplementary material:**

The online version of this article (doi:10.1007/s11751-014-0187-x) contains supplementary material, which is available to authorized users.

## Introduction

The majority of tibial fractures go on to uneventful union. Non-unions are reported in 6.95 % in one series [1]. The precise definition of non-union is difficult, and arbitrary time limits of 9 months from injury or 3 months without radiographic progression of healing have been proposed [2]. A more practical definition is a fracture that has failed to heal within the expected time, given the individual fracture characteristics.

The aetiology of non-union includes factors relating to the patient, the injury pattern and previous interventions. Successful treatment requires each of these areas to be addressed. In fractures involving severe bone and soft tissue injury, bone loss or significant deformity, the use of circular fine wire fixators is an important part of the surgical armamentarium. They allow concomitant correction of deformity and bone loss with resolution of non-union while minimising soft tissue disruption. In a series of tibial non-unions, including those with significant bone loss, union was achieved in 93 % with the use of circular fixators [3].

The excellent potential surgical outcomes that can be achieved with circular fixators must be offset against personal cost to the patient. Treatment is often prolonged with a substantial amount of time spent in a cumbersome, at times painful, frame. Problems can arise due to pin-site infection and soft tissue tethering. This has a negative impact on both work and home life.

The purpose of this study was to obtain patient-reported outcomes in patients with tibial non-unions who were successfully treated with circular fixators.

## Materials and methods

From a prospective database at our institution, we identified twenty-one patients who had undergone limb reconstruction for tibial non-union using a circular frame. The patients were treated by the senior author and have the same support from clinicians and peers through attendance at the same weekly dedicated physiotherapy class.

Each patient was sent a postal questionnaire based on the Enneking scoring system and the Euroqol EQ5D outcome score. Enneking described a scoring system for use in patients undergoing limb salvage surgery for bone tumours [4]. The score consists of subjective reporting of pain, function, gait and impairment together with an evaluation of the patient’s attitudes and emotional acceptance of their treatment. A percentage score is calculated on the sum of the scores for each question divided by the maximum possible score. Given that limb reconstruction surgery can involve prolonged complicated treatment similar in intensity to oncological limb salvage, its parallel use in assessing the management of complex trauma is endorsed in the current BOA/BAPRAS guidelines on open tibial fractures [5].

The euroqol questionnaire is a general health questionnaire that has been validated for the UK population [6]. It assesses pain, mobility, self-care, anxiety and ability to perform usual activities. In addition, a visual analogue scale (VAS) is used to quantify the patients’ own assessment of their current health state, with 0 the worst possible and 100 the best possible health. Age and sex means for the VAS have been published [7], which allow the calculation of a *z*-score defined as the expected score by age and sex subtracted from the patients score and divided by the population standard deviation.

The radiographs of all patients were reviewed to detail the initial injury which was classified using the AO system. The operative details and time in frame were also noted.

## Results

Replies were received from 14 patients with a mean age of 48.2 years (21–67 years). There were 3 females. The commonest original fracture pattern was 42-B, affecting 5 fractures, with three 42-A- and three 42-C-type fractures, and two 41-C- and one 43-A-pattern fractures. Four patients were smokers at the time of referral. Six fractures were complicated by infection. All fourteen cases went on to achieve union. The mean time in a frame was 10.1 months (4–20 months). Figures (Supplementary material Figs. 1, 2, 3) illustrate the treatment of a patient with an infected non-union and Figures (Supplementary material Figs. 4, 5, 6) the treatment of an aseptic non-union.

Complications during treatment included four patients with pin-site infections, which were managed with antibiotics. In addition, there were two cases of premature consolidation of regeneration, necessitating a repeat osteotomy.

The Enneking score ranged from 34.3 to 77.1 % with a mean of 58 %. In terms of emotional acceptance of the treatment, two patients reported being enthused by their treatment, 4 intermediate between enthused and satisfied, 4 satisfied, 3 intermediate between satisfied and accepting, and one accepting of the treatment. Three patients stated they would not go through the same treatment again.

The Euroqol questionnaire results showed that 8 patients had some difficulty with mobility, 10 had some difficulty with usual activities, and 12 reported moderate pain.

The overall mean score on the VAS of general health state was 77.1 (48–90), and the mean predicted for an age- and sex-matched UK population was 83.1 (70.7–87.3). There was no statistically significant difference between the two groups. In six patients, the reported score was in fact higher than the predicted score, and all but three fell within one standard deviation of the predicted score.

Comparing the infected and aseptic non-union groups, there was no significant difference in time in frame or the Euroqol VAS z-score. There was a statistically significant difference in the Enneking score between the aseptic (mean 65.3, range 45.7–77.1) and infected (mean 48.1, range 34.3–65.7) groups (*p* = 0.013, independent *t* test).

## Discussion

Successful treatment of tibial non-union can be achieved in a variety of ways. The method chosen will depend on injury factors, previous treatments, the presence of infection, bone loss and limb alignment; these are considered with the acceptability of the proposed treatment strategy to the patient.

In fractures complicated by infection, significant bone loss or malalignment, the use of circular frames allows all these to be addressed concurrently while treating the non-union. Mahaluxmivala et al. treated 18 non-unions, including 10 infected cases, with a circular frame. All patients went on to achieve union. Six patients underwent bone transport for bone loss, five of whom required additional bone grafting [8]. Hosny et al. [9] used circular frames and a compression–distraction technique to treat eleven infected non-unions; eight patients achieved a good or excellent result. Dujardyn et al. [10] reported union in 27 of 28 patients managed with an Ilizarov frame and partial fibulectomy; they found smoking adversely affected healing time. In their paper, Brinker et al. [11] treated patients over 60 years of age with circular frame, achieving union in all 20 patients who completed treatment, with an improvement in quality of life equivalent to 5.3 quality-adjusted years. Rozbruch et al. treated 38 non-unions with a Taylor Spatial Frame achieving union in 27 patients; the remaining eleven required further procedures including 2 amputations. Good or excellent functional results were reported in 34 patients [12].

Despite the good clinical outcomes that can be achieved, living with a frame can result in significant morbidity. Four of fourteen patients in this report had problems with infected pin sites. Foster et al. [13] in their paper reported a 23 % rate of pin-site infection in 40 complex tibial fractures treated with a circular frame. Saw et al. [14] reported a 6 % rate of pin-site infection with increased rates in half pins and distally sited wires. Other complications of circular frames include pain, soft tissue tethering and heel cord tightness. Garcia-Cimbrelo et al. [3] reported heel cord tightness requiring Achilles tendon lengthening in 11 from 82 patients undergoing treatment of tibial non-union with increased rates in those having concomitant treatment of bone defects.

The psychosocial effects of living with a circular external fixator are difficult to quantify although living with a frame undoubtedly affects all aspects of a patient’s life. In a study of adolescents treated with circular frames, Martin et al. [15] found that patients reported living with a frame to be better than anticipated and emphasised the importance of peer support.

Our study is a small retrospective review of a single-surgeon series of patients with the inherent limitations of such a study. We achieved a good response rate from our group of patients. We intentionally used scoring systems that allowed patients to make subjective assessments of their treatment but acknowledge a potential bias in patients who have undergone successful treatment.

There is an increasing emphasis within orthopaedics on the use of patient-reported outcome scores. We found the Enneking scoring system to be relevant to our population of patients and would recommend the use of such a scoring system in limb reconstruction. We found that all patients were accepting their treatment with a small number enthused by it, all but three would not go through the same treatment again. Despite the fact that most patients experienced a varying degree of pain and limitation in daily activities, we found no difference in overall health state compared with an age- and sex-matched UK population.

Circular frames are undoubtedly a valuable tool in the treatment of tibial non-union, particularly those complicated by malalignment, infection or bone loss. The experience can be arduous for the patient, and effective pre-operative counselling regarding the experience of living in a frame and the likely clinical outcome are essential to allow patients to make informed decisions. Using patient-reported outcomes in pre-operative discussions, surgeons can help to quantify anticipated outcomes following treatment and facilitate the decision-making process for their patients.

## Electronic supplementary material

Below is the link to the electronic supplementary material.

Supplementary material 1 (JPEG 193 kb)

Supplementary material 2 (JPEG 399 kb)

Supplementary material 3 (JPEG 166 kb)

Supplementary material 4 (JPEG 182 kb)

Supplementary material 5 (JPEG 281 kb)

Supplementary material 6 (JPEG 187 kb)
