# Supplementary figures and images for: Patient-reported outcomes following treatment of tibial non-union with circular frames
Source: Strategies Trauma Limb Reconstr. 2014 Feb 12;9(1):33–5. doi: 10.1007/s11751-014-0187-x (PMC3951624; doi:10.1007/s11751-014-0187-x)

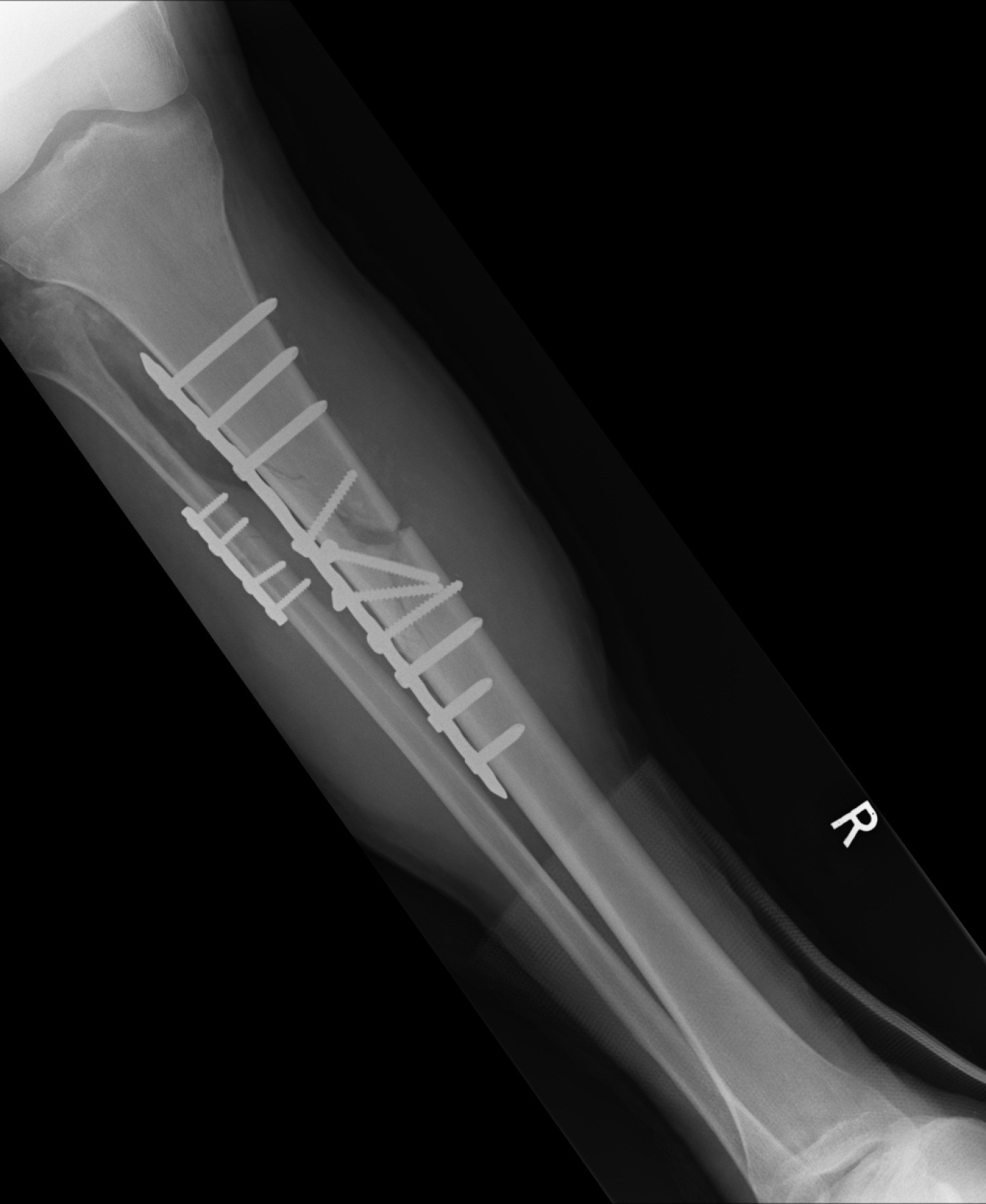

Supplement: Supplementary file 1 — Supplementary material 1 (JPEG 193 kb) [file 11751_2014_187_MOESM1_ESM.jpg]

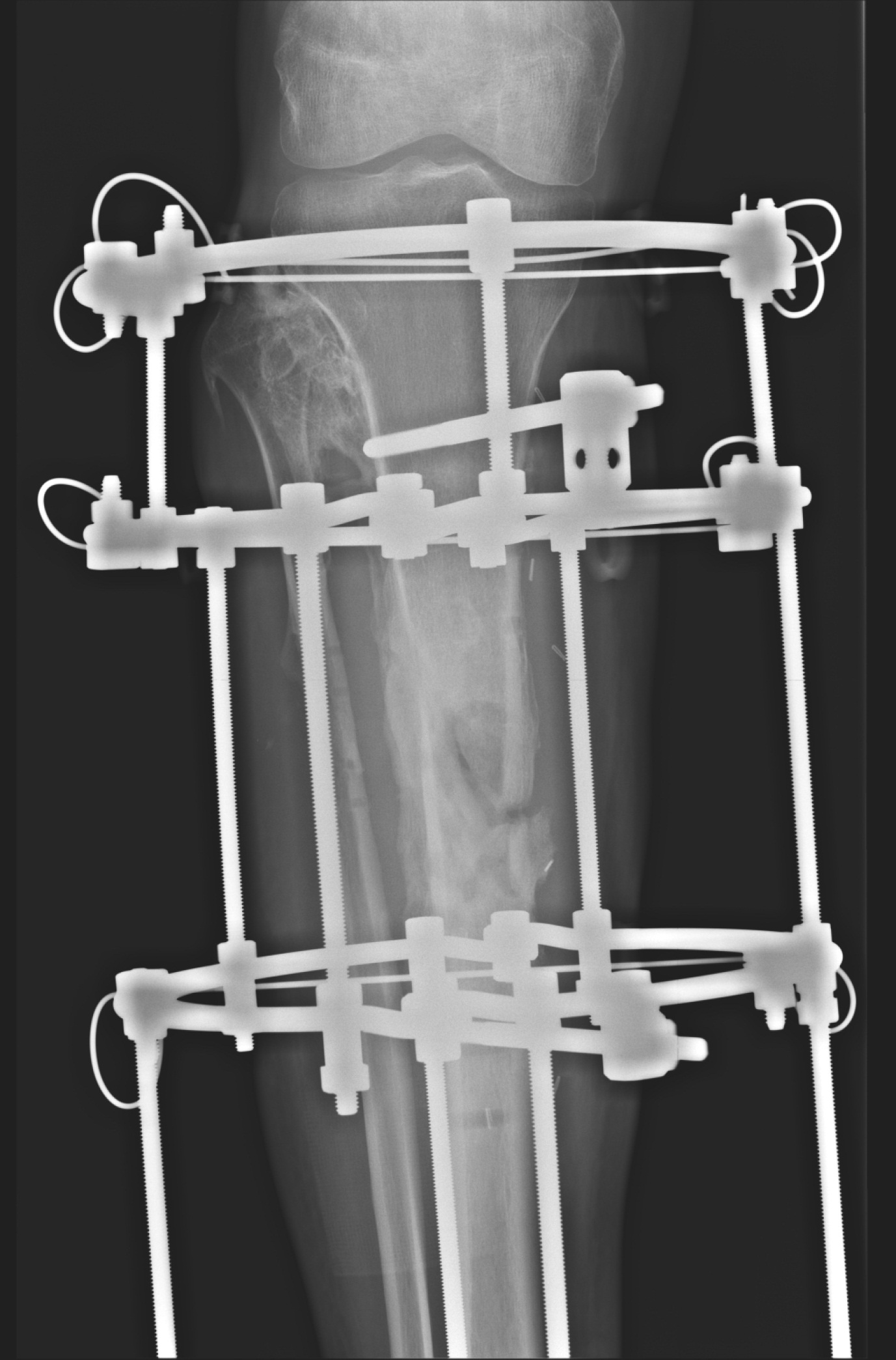

Supplement: Supplementary file 2 — Supplementary material 2 (JPEG 399 kb) [file 11751_2014_187_MOESM2_ESM.jpg]

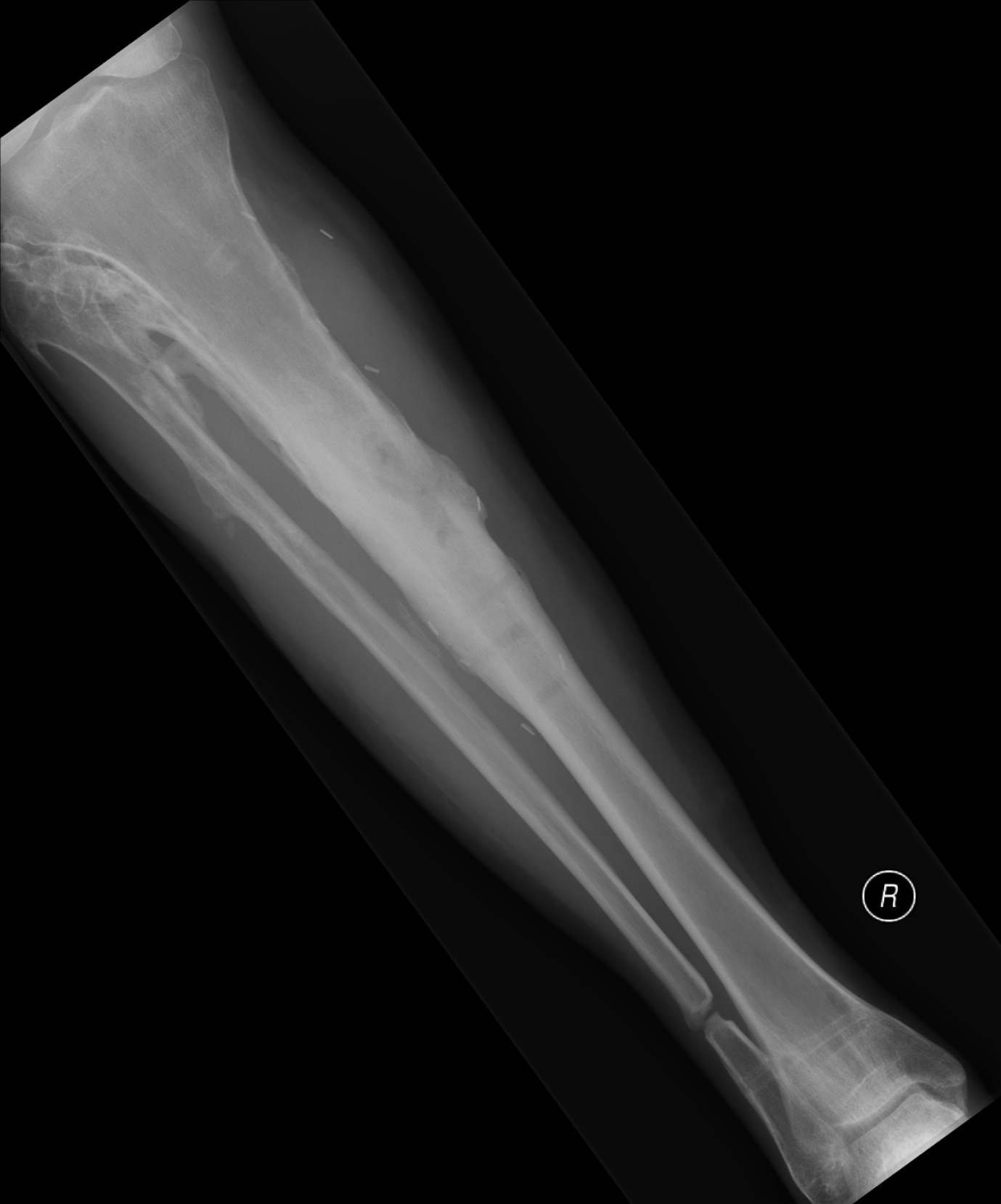

Supplement: Supplementary file 3 — Supplementary material 3 (JPEG 166 kb) [file 11751_2014_187_MOESM3_ESM.jpg]

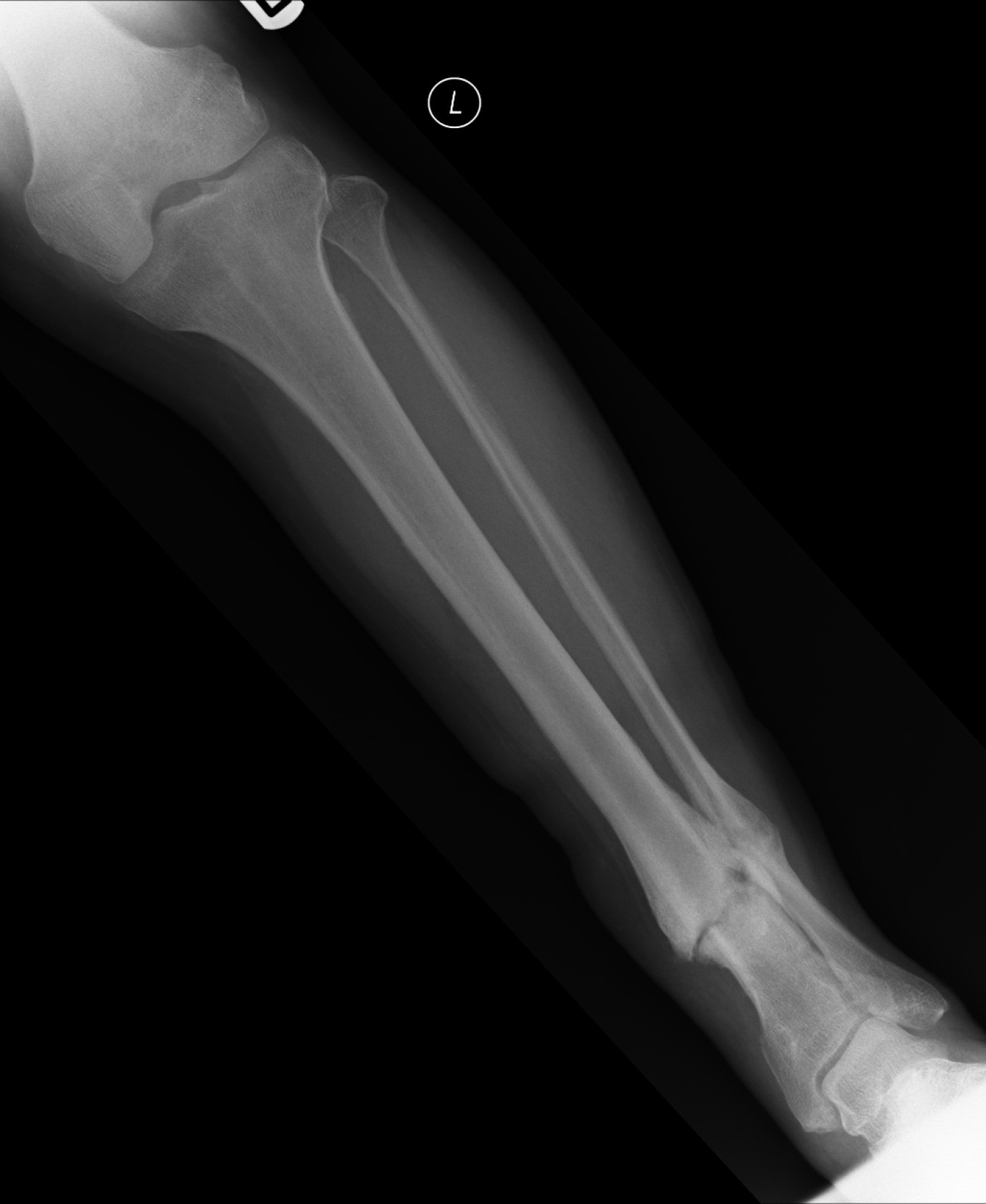

Supplement: Supplementary file 4 — Supplementary material 4 (JPEG 182 kb) [file 11751_2014_187_MOESM4_ESM.jpg]

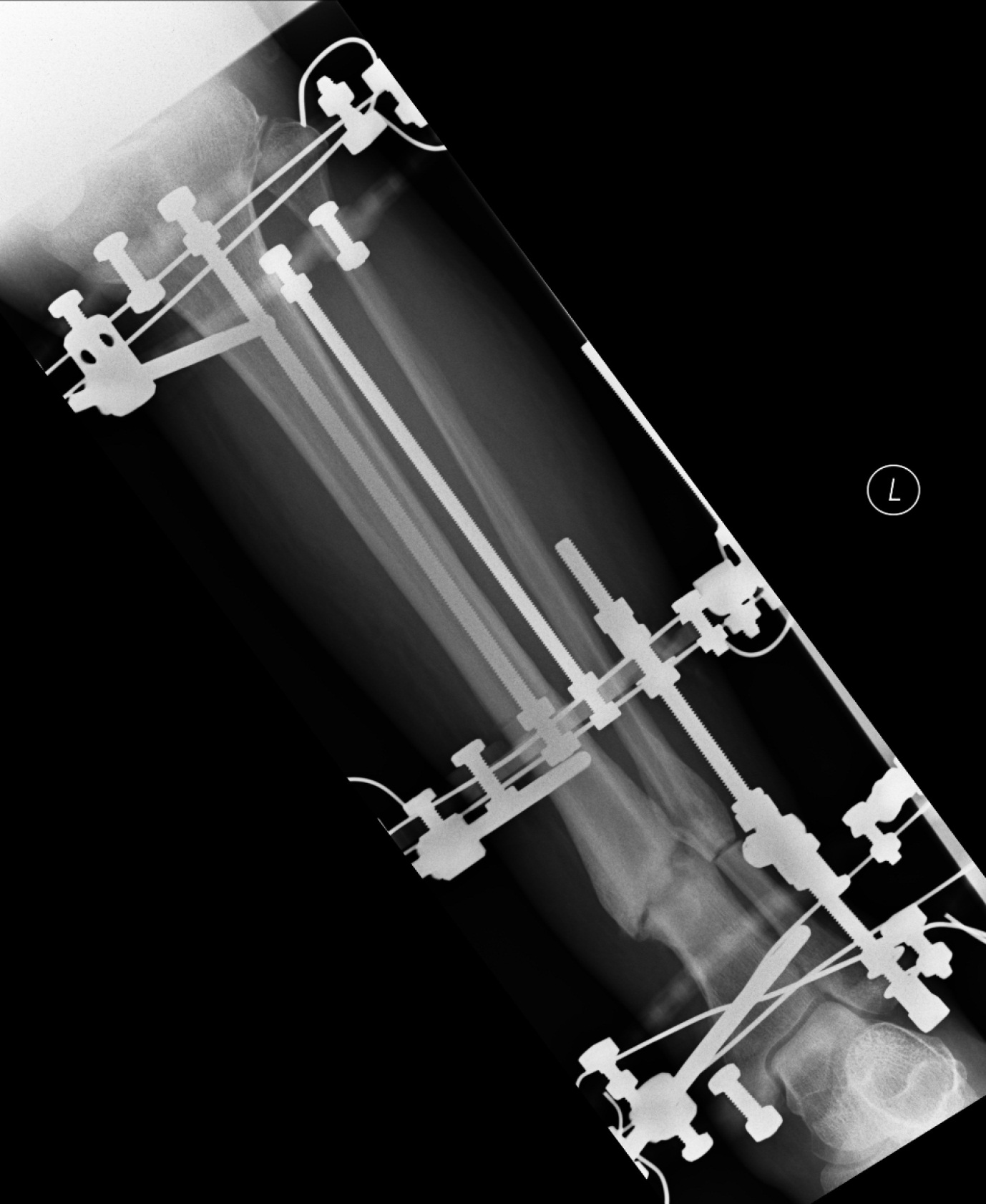

Supplement: Supplementary file 5 — Supplementary material 5 (JPEG 281 kb) [file 11751_2014_187_MOESM5_ESM.jpg]

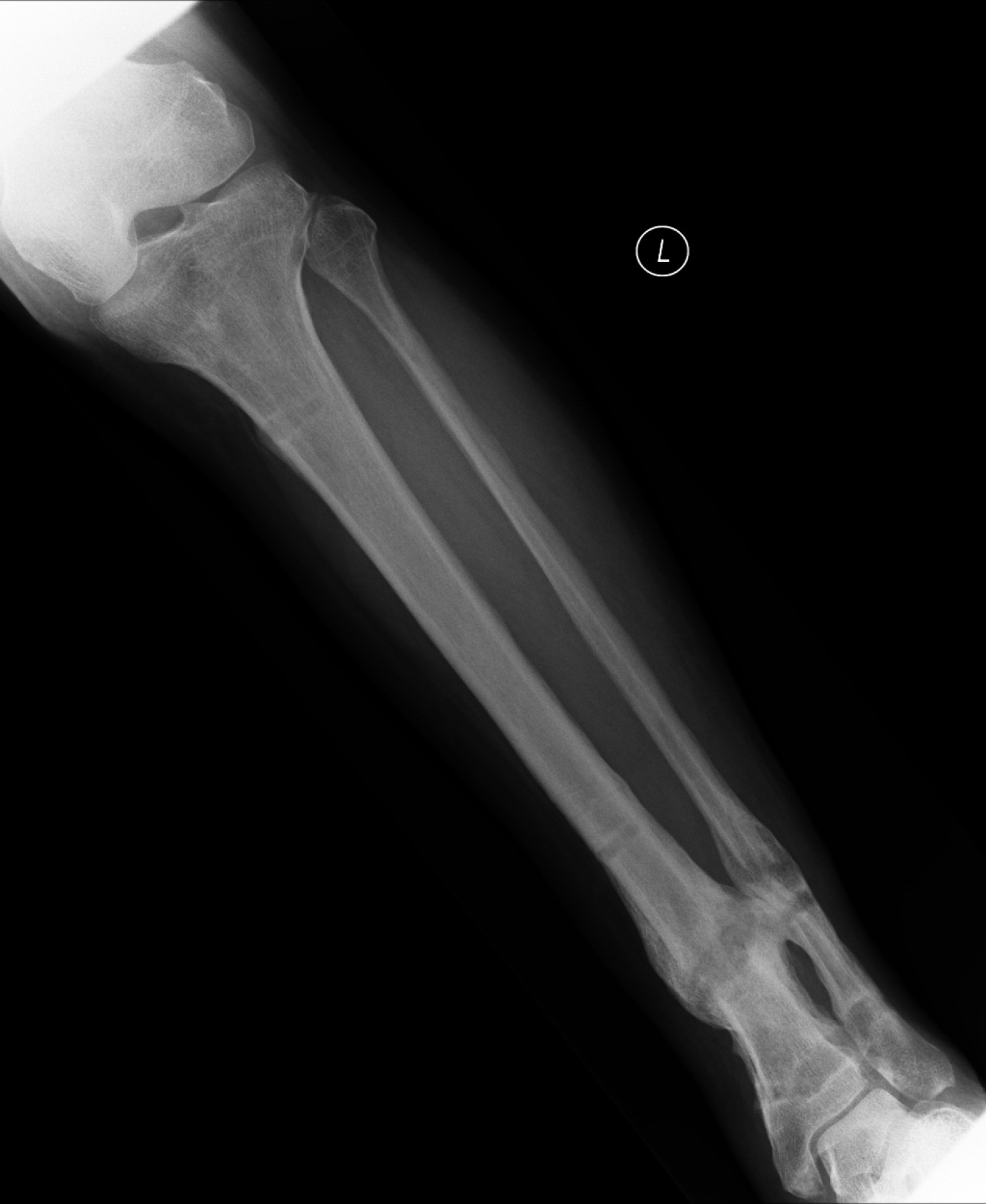

Supplement: Supplementary file 6 — Supplementary material 6 (JPEG 187 kb) [file 11751_2014_187_MOESM6_ESM.jpg]
